# Of mice and humans: how good are HLA transgenic mice as a model of human immune responses?

**DOI:** 10.1186/1745-7580-5-3

**Published:** 2009-06-17

**Authors:** Maya F Kotturi, Erika Assarsson, Bjoern Peters, Howard Grey, Carla Oseroff, Valerie Pasquetto, Alessandro Sette

**Affiliations:** 1 Division of Vaccine Discovery, La Jolla Institute for Allergy and Immunology, 9420 Athena Circle, La Jolla, CA 92037, USA

## Abstract

**Background:**

Previous studies have defined vaccinia virus (VACV)-derived T cell epitopes in VACV-infected human leukocyte antigen-A*0201 (HLA-A2.1) transgenic (Tg) mice and A2.1-positive human Dryvax vaccinees. A total of 14 epitopes were detected in humans and 16 epitopes in A2.1 Tg mice; however, only two epitopes were independently reported in both systems. This limited overlap raised questions about the suitability of using HLA Tg mice as a model system to map human T cell responses to a complex viral pathogen. The present study was designed to investigate this issue in more detail.

**Results:**

Re-screening the panel of 28 A2.1-restricted epitopes in additional human vaccinees and in A2.1 Tg mice revealed that out of the 28 identified epitopes, 13 were detectable in both systems, corresponding to a 46% concordance rate. Interestingly, the magnitude of responses in Tg mice against epitopes originally identified in humans is lower than for epitopes originally detected in mice. Likewise, responses in humans against epitopes originally detected in Tg mice are of lower magnitude.

**Conclusion:**

These data suggest that differences in immunodominance patterns might explain the incomplete response overlap, and that with limitations; HLA Tg mice represent a relevant and suitable model system to study immune responses against complex pathogens.

## Background

Model systems to study human responses to potential pathogens in general, and to complex viruses such as poxviruses in particular, are of considerable practical interest. They allow for characterization of immune responses directed against epitopes potentially capable of being recognized in humans in a rigorously controlled experimental situation, without having to rely on samples derived from exposed individuals. These issues are particularly relevant in the case of pathogens of bioterrorism concern, as well as emerging and re-emerging pathogens.

Several alternative approaches have been described to circumvent the need to use samples from infected humans. These include using mice re-colonized with human hematopoietic stem cells, primary *in vitro *restimulation utilizing peripheral blood mononuclear cells (PBMC) from healthy volunteers, and human leukocyte antigen (HLA) transgenic (Tg) mice. Murine/human chimeric systems appear to be associated with significant experimental variability, and responses of low magnitude and diversity compared to those observed in humans have been reported [1]. Primary *in vitro *restimulation systems are an attractive alternative, but are associated with limited throughput, high cost, labor intensiveness, and the lack of testing the impact of variables associated with host-pathogen interactions in a living organism.

Several groups have reported the development and validation of HLA Tg mice as a model system to study T cell responses restricted by human major histocompatibility complex (MHC) molecules [2-4]. It has been established that HLA Tg mice represent a fairly accurate model of human immune responses based on peptide immunizations. For example, Wenthworth and co-workers analyzed the repertoire of influenza-specific T cell responses obtained in HLA Tg mice with that obtained following primary induction of human cytotoxic T lymphocyte (CTL), and observed concordant results for approximately 85% of the peptides assayed [4].

The question of how suitable HLA Tg mice are for defining responses to complex pathogens and antigens has, however, not been addressed in detail. This issue is relevant because significant differences between humans and HLA Tg mice could exist at several levels. It is possible that co-expression of different MHC molecules might impact the TCR repertoire, in that certain specificities are lost while others are gained, as a function of the MHC alleles present [5]. Differences also exist in the cellular processing machinery operating in mice and humans at the level of the proteasome and TAP transport [6-8]. Discrepancies might also exist in the pattern of expression of viral antigens in infected cells of human and murine origin. Furthermore, it is currently unknown to what degree these differences might influence the global outcome of immune responses.

In our laboratory, we have characterized the T cell response to VACV observed in infected HLA-A*0201 (A2.1) Tg mice [5] and in human Dryvax vaccinees expressing A2.1 [9]. This enabled us to compare responses to a complex viral pathogen in the two different systems. We have identified a total of 28 different A2.1-restricted epitopes, and other investigators have described three additional epitopes (J8R_11–19_, A47L_169–177_, and B5R_5–19_) using A2-positive vaccinees [10-12]. Concordant results were found in some cases, but for the most part, epitopes detected in humans were not detected in mice, and vice versa. In the present study, we have further assessed the issue of limited epitope overlap observed in humans and HLA Tg mice.

## Results and discussion

### Additional testing in A2.1 Tg mice reveals a more extensive repertoire overlap

**Figure 1:**
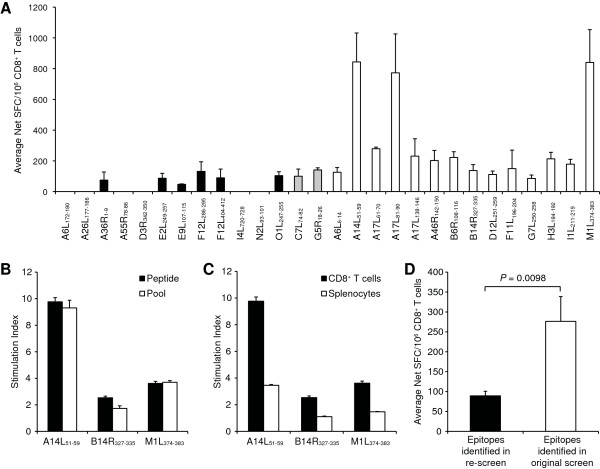
**Testing A2.1-restricted VACV T cell epitopes in A2.1 Tg mice using purified CD8^+ ^T cells and individual peptides**. (A) Splenic CD8^+ ^cells were purified from VACV-infected A2.1 Tg mice and incubated with peptide-pulsed Jurkat-A2.1/K^b ^cells, after which number of IFN-γ-producing cells was enumerated in an ELISPOT assay. Shown are the average net SFC/10^6 ^CD8^+ ^T cells in response to each peptide; black bars, epitopes identified by Oseroff *et al. *[9] in A2.1 positive donors and recognized in Tg mice upon re-screening; white bars, peptides identified by Pasquetto *et al. *[5] in Tg mice; grey bars, epitopes identified in both studies. (B) The specific T cell response to three different epitopes (B14R_327–335_, M1L_374–383_, and A14L_51–59_) was assessed 7 days after VACV infection in A2.1 Tg mice. Shown is the stimulation index for the epitopes tested either as individual peptides (black bars) or in pools of ten peptides (white bars). (C) Shown is the stimulation index for the epitope-specific responses by using either CD8^+ ^T cells (black bars) or splenocytes (white bars). (D) Shown are the average net SFC/10^6 ^CD8^+ ^T cells in response to groups of epitopes recognized in Tg mice upon re-screening (black bar) and identified in Tg mice in the original study by Pasquetto *et al. *[5] (white bar). The unpaired t test with Welch's correction was used to determine if differences were significant. For all panels, average data are shown from at least two independent experiments. For each individual experiment samples are tested in triplicate. Error bars indicate SEM.

We previously detected 14 different epitopes utilizing PBMC from A2.1-positive human volunteers vaccinated with Dryvax (Figure 1A) [9]. Of these epitopes, only two were also independently identified in VACV-infected A2.1 Tg mice [5,13-16]. Based on these results, Terajima and Ennis hypothesized that the large number of potentially immunogenic peptides encoded by a complex pathogen such as VACV might explain the incomplete overlap observed [17]. Herein, we addressed this issue in more detail.

First, A2.1 Tg mice were inoculated with VACV and the reactivity against each of the 28 individual A2.1-restricted epitopes was evaluated. Responses were detected for all epitopes previously reported in HLA Tg mice (Figure 1A; white and grey bars). In addition, responses were detected for 6 peptides that had been originally described in A2.1-positive humans [9,14,18], but not reported in studies utilizing Tg mice [5,15] (Figure 1A; black bars). Altogether, responses directed against 8 of 14 (57%) epitopes reported in humans were now also detected in A2.1 Tg mice.

The present epitope screen differs in two experimental settings from the original study by Pasquetto *et al. *[5]. First, individual peptides were used here instead of peptide pools. Second, the initial epitope identification used splenocytes as effector cells [5], whereas the present study utilized purified CD8^+ ^T cells. Herein we examined the impact of these two experimental differences. When three representative epitopes (A14L_51–59_, B14R_327–335_, and M1L_374–383_) were tested either as individual peptides or in pools of 10 peptides, similar response magnitudes were observed (Figure 1B). We also tested the response to the same 3 epitopes using either purified CD8^+ ^T cells or splenocytes, and found that the higher non-specific interferon (IFN)-γ production obtained when using splenocytes led to a reduced stimulation index, and thereby decreased sensitivity (Figure 1C).

We observed that the average responses against these epitopes not initially identified in Tg mice were ~3 times lower than those against the epitopes identified in the original screen (*P *= 0.0098; Figure 1D). Although the 3 dominant epitopes (A14L_51–59_, A17L_81–90_, and M1L_347–383_) contribute to a significant portion of the higher response identified in mice, exclusion of these 3 epitopes still results in a significant difference in the magnitude of the response for the epitopes identified in humans versus mouse (*P *= 0.0012). Taken together, these results suggest that while 8 of 14 (57%) peptides reported in humans can also be detected in A2.1 Tg mice, differences in the patterns of immunodominance might render them less prominent and consequently make their detection more difficult.

The mice utilized by Pasquetto *et al*. were A2.1 bxd mice [5]. To examine whether expression of endogenous mouse MHC molecules might also contribute to the incomplete overlap, we tested the reactivity of the 14 epitopes identified in humans using CD8^+ ^T cells from VACV-infected HHD A2.1 mice, which do not express endogenous mouse MHC [19]. However, no additional epitopes were recognized in the HHD mice, and the response magnitude and immunodominance pattern was similar to that in A2.1 bxd mice (data not shown).

### Testing of a broader panel of human vaccinees reveals a more extensive overlap

As described above, a total of 16 epitopes were initially identified in HLA Tg mice [5,13,15]. Of these, only two (13%) were also independently identified in human vaccinees (Figure 1A). Most of the candidate peptides were tested in only 6 donors, with each individual donor yielding a rather distinct pattern of epitope recognition. While some epitopes were identified in multiple donors, most of the epitopes were only detected in 1 out of the 6 donors investigated [9].

To test the hypothesis that the epitopes not initially detected in humans were associated with lower and/or less frequent responses, we examined the responses to the set of 16 A2.1-restricted epitopes identified in Tg mice using PBMC from 8 different A2-positive vaccinees. The samples tested included frozen cell aliquots from 5 of the donors tested in the original study of Oseroff *et al. *[5], as well as PBMC from 3 additional A2-positive vaccinees. PBMC were cultured in the presence of individual peptides, and the numbers of IFN-γ-producing cells were enumerated in an enzyme-linked immunosorbent spot (ELISPOT) assay. The same two peptides previously identified as positive in these donors were also re-identified in the course of the present experiments, and responses to an additional 5 peptides originally identified in A2.1 Tg mice, but not identified in the human donor screen of Oseroff *et al. *[9], were detected. Similar to what we observed in the mouse experiments, responses to the newly detected epitopes were in general lower than to epitopes identified in the initial screen (see Additional file 1). Overall, responses directed against 7 of 16 (44%) peptides initially identified in HLA Tg mice, were also detected in humans. Considering the large donor-to-donor variation observed, it is likely that testing of additional donors would further increase the overlap between the two systems.

## Conclusion

In summary, of the 28 epitopes identified in either humans or mice, in this study we showed that 13 were detected in both systems, with an overall concordance rate of 46% (Table 1). This degree of overlap is, albeit incomplete, much greater than previously detected. In a similar study, a 68% concordance rate was observed between human immunodeficiency virus type 1 (HIV-1)-specific CD8^+ ^T cell epitopes recognized in both HLA-A2.1 Tg mice and HLA-A2 HIV-1 positive patients [20]. A higher degree of overlap was likely detected because HIV-1 epitope recognition in A2.1 Tg mice was evaluated following peptide immunization and not natural infection, a larger cohort of donors were tested, and HIV-1 has a significantly smaller genome than VACV. These data suggest that while the Tg mouse system is suitable to model immune responses to complex pathogens, caution should be taken in interpreting the results obtained.

**Table 1:** Summary of overlap between A2.1-restricted VACV T cell epitope recognition in HLA Tg mice and human systems

**Epitope**	**Sequence**	**Original Identification**	**Reference**	**Average** **Net SFC/10^6 ^Cells**	
				
				**Tg Mice**	**Human**
A6L_172–180_	ILSDENYLL	Human	[9]	-	106
A26L_177–186_	YLYTEYFLFL	Human	[9]	-	164
A36R_1–9_	MMLVPLITV	Human	[9]	75	49
A55R_78–86_	YIYGIPLSL	Human	[9]	-	94
D3R_342–350_	FLVIAINAM	Human	[9]	-	195
E2L_249–257_	KIDYYIPYV	Human	[9]	87	192
E9L_107–115_	FLNISWFYI	Human	[9]	48	132
F12L_286–295_	NLFDIPLLTV	Human	[9]	132	252
F12L_404–412_	FLTSVINRV	Human	[9]	90	73
I4L_720–728_	SMHFYGWSL	Human	[9]	-	102
N2L_93–101_	YVNAILYQI	Human	[9]	-	563
O1L_247–255_	GLNDYLHSV	Human	[9]	104	77

C7L_74–82_	KVDDTFYYV	Human + Tg mice	[5,9,14,15,18]	100	178
G5R_18–26_	ILDDNLYKV	Human + Tg mice	[5,9,14]	141	271

A6L_6–14_	VLYDEFVTI	Tg mice	[5]	126	-
A14L_51–59_	FILGIIITV	Tg mice	[5]	844	60
A17L_61–70_	RTLLGLILFV	Tg mice	[5]	279	-
A17L_81–90_	ILMIFISSFL	Tg mice	[5]	773	42
A17L_138–146_	QIFNIISYI	Tg mice	[13]	231	-
A46R_142–150_	GLFDFVNFV	Tg mice	[5]	202	-
B6R_108–116_	LMYDIINSV	Tg mice	[5]	222	-
B14R_327–335_	HVDGKILFV	Tg mice	[5]	137	-
D12L_251–259_	RVYEALYYV	Tg mice	[5]	112	63
F11L_196–204_	FLIVSLCPT	Tg mice	[5]	150	-
G7L_250–258_	YLPEVISTI	Tg mice	[5]	86	-
H3L_184–192_	SLSAYIIRV	Tg mice	[5,16]	213	-
I1L_211–219_	RLYDYFTRV	Tg mice	[5]	179	115
M1L_374–383_	IIIPFIAYFV	Tg mice	[5]	840	91

Despite the more extensive and sensitive system used in the current study, approximately one half of the epitopes recognized specifically in one system were not recognized in the other. Differences in TCR repertoire between mouse and human might account for the different epitope recognition. However, peptide immunizations of the human epitopes in A2.1 Tg mice only failed in 2 of 14 instances to generate a T cell response. This is similar to the 85% success rate previously reported by Wentworth *et al. *[4], and suggests that repertoire differences account for a relatively minor percentage of the discrepancies. Differences in the processing apparatus and antigen presentation in murine versus human dendritic cell (DC) subsets and other antigen-presenting cells (APCs) involved in priming CTL immunity might also explain the differential recognition of some epitopes [7]. In mice, multiple DC subsets have been described, however, CD8^+ ^DCs are considered the primary DC subset involved in directly activating naïve CD8^+ ^T cells during VACV infection [21]. Recently, the CD8^- ^DC subset has also shown to play an important role [22]. In humans, various cell types are susceptible to VACV infection, including dermal DCs, Langerhans cells, and macrophages [23,24], but the primary APC responsible for naïve CD8^+ ^T cell priming is unknown. As the peptides identified in the Tg mice were effectively processed in VACV-infected human APCs [5], differences in processing appear an unlikely explanation. Furthermore, while mice were immunized i.p. and humans were vaccinated by dermal scarification, we recently reported that either route generates similar T cell responses, and only minor differences in magnitude of responses were observed in mice [25].

Overall, our data suggest that while about half of the epitopes are recognized both in humans and Tg mice, the actual magnitudes vary, and thus differences in the immunodominance pattern contribute to the degree of overlap in the responses observed. Discrepancies in the kinetics of viral antigen expression in infected cells of human and murine origin might impact immunodominance [26]. Interestingly, a recent study mapping HLA-A2.1-restricted epitopes derived from modified VACV Ankara-infected human B cells by differential HPLC-coupled mass spectrometry found epitopes predominantly derived from early gene products [27]. However, we identified A2.1-restricted epitopes in HLA Tg mice and human vaccinees from gene products expressed both early and late during the viral life cycle [28]. Despite having comparable T cell repertoires, the differences in immunodominance might be due to distinct naïve CD8^+ ^T cell precursor frequencies in the two systems. We have recently demonstrated that precursor frequencies shape the CD8^+ ^T cell immunodominance hierarchy following LCMV infection [29], and therefore this might also apply to a more complex virus, such as VACV. These conclusions are in agreement with those drawn from a recent study mapping the T cell responses in VACV-immune individuals, where it was pointed out that an epitope might be considered immunodominant if recognized by both humans and HLA Tg mice [30]. In summary, the present study suggests that, with limitations, HLA Tg mice represent a relevant and suitable model system for identification and validation of T cell epitopes recognized during the course of complex viral infection in humans.

## Methods

### Peptides

Peptides were synthesized by A and A Labs and purified to >95% homogeneity by reverse-phase high-performance liquid chromatography (HPLC). Purity was determined using analytical reverse-phase HPLC and amino acid analysis, sequencing, and/or mass spectrometry. Peptides were radio-labeled with the chloramine T method as described [31].

### Characteristics of study population

Healthy males and females between 18 and 59 years of age were used in this study. Exclusion criteria were body weight of < 45.4 kg and established pregnancy. Recruited donors were vaccinated by arm scarification with a VACV (Dryvax) vaccination as a prophylactic measure either because of their potential exposure to VACV in a laboratory or hospital setting or because of their enrollment into military and health worker vaccination program. The blood draw was performed within 1 year of the Dryvax vaccination. All experiments carried out with human subjects were in compliance with the Helsinki Declaration. Institutional Review Board approval (FWA# 00000032) and appropriate informed consent were obtained for this study.

### PBMC isolation and HLA typing

PBMC were isolated from heparinized blood by gradient centrifugation with a Histopaque-1077 (Sigma-Aldrich, St. Louis, MO) [32], suspended in fetal bovine serum containing 10% dimethyl sulfoxide, and cryo-preserved in liquid nitrogen. Donor's PBMC were typed for HLA-A by high-resolution polymerase chain reaction (Forensic Analytical Molecular Genetics, San Francisco, CA).

### Mice

A2.1 Tg mice used in this study were the F_1 _generation derived from crossing homozygous Tg mice (H-2^b ^haplotype) expressing a chimeric gene (A2.1/K^b^) consisting of the α1 and α2 domains of HLA and the α3 domain of H-2K^b ^with BALB/c mice (The Jackson Laboratory, Bar Harbor, ME) [4,33,34]. The HHD A2.1 strain [19] was kindly provided by Dr. Jack Bennink (NIAID, NIH, Bethesda, MD). All mice were bred and maintained following NIH guidelines and Institutional Animal Care and Use Committee-approved animal protocols (AAALAC# 000840 and OLAW# A3779-01).

### Viruses and infection

The Western Reserve strain of VACV was obtained from Dr. Bernard Moss (NIAID). Mice were infected i.p. with 2 × 10^6 ^PFU of VACV. On day 7 post-infection, the mice were sacrificed and splenic CD8^+ ^T cells were used in mouse IFN-γ ELISPOT assays.

### *Ex vivo *IFN-γ ELISPOT

IFN-γ ELISPOT assays were performed as described [5,9]. In brief, for murine assays, 2 × 10^5 ^splenic CD8^+ ^T cells were cultured with 10^5 ^human Jurkat cells (expressing the same A2.1/K^b ^construct as in the Tg mice) pulsed with 10 μg/ml of peptide. For human assays, 2 × 10^5 ^PBMC were incubated with 5 μg/ml of peptide. After a 20 h incubation at 37°C, plates were developed, and responses calculated as described [5,9,19]. Criteria for positivity were net spot-forming cells (SFC)/10^6 ^cells ≥ 20, stimulation index ≥ 2.0, and p-value ≤ 0.05 using a Student's *t *test in at least 2 out of 3 experiments.

## Abbreviations

A2.1: HLA-A*0201; APC: antigen-presenting cell; CTL: cytotoxic T lymphocyte; DC: dendritic cell; ELISPOT: enzyme-linked immunosorbent spot; HIV-1: human immunodeficiency virus type 1; HLA: human leukocyte antigen; HPLC: high-performance liquid chromatography; IFN-γ: interferon-γ; MHC: major histocompatibility complex; PBMC: peripheral blood mononuclear cells; SFC: spot-forming cells; Tg: transgenic; VACV: vaccinia virus.

## Competing interests

The authors declare that they have no competing interests.

## Authors' contributions

MFK, EA, CO, and VP performed the human and mouse immunoassays. AS conceived the study. BP and HG aided with data analysis. MFK, EA, and AS wrote the manuscript. All authors participated in discussions, and reviewed and approved the final manuscript version.

## Supplementary Material

Additional file 1 **Recognition of additional A2.1-restricted VACV T cell epitopes by human A2-positive VACV vaccinees**. The data provided represent the net SFC/10^6 ^PBMCs of the human A2-positive VACV vaccinees in response to epitopes originally identified in A2.1 Tg mice. Click here for file
