# Supplementary material for: Of mice and humans: how good are HLA transgenic mice as a model of human immune responses?
Source: Immunome Res. 2009 Jun 17;5:3. doi: 10.1186/1745-7580-5-3 (PMC2702351; doi:10.1186/1745-7580-5-3)
Supplement: Additional file 1 — Recognition of additional A2.1-restricted VACV T cell epitopes by human A2-positive VACV vaccinees. The data provided represent the net SFC/106 PBMCs of the human A2-positive VACV vaccinees in response to epitopes originally identified in A2.1 Tg mice. [file 1745-7580-5-3-S1.doc]

**Additional file 1.** Recognition of additional A2.1-restricted VACV T cell epitopes by human A2-positive VACV vaccinees.

| **Epitope** | **Sequence** | **Original Identification** | **Human Vaccinees** | | | | | | | | **#Responders (out of 8)** |
| --- | --- | --- | --- | --- | --- | --- | --- | --- | --- | --- | --- |
|  |  |  | **V103^a^** | **V111^a^** | **V145** | **V148^a^** | **V155** | **V157^a^** | **V171** | **V172^a^** |  |
| A6L_6-14_ | VLYDEFVTI | Tg mice | 9 ^b^ | -3 | 0 | 3 | 8 | -2 | 38 | -3 | 0 |
| A14L_51-59_ | FILGIIITV | Tg mice | 19 | 0 | 7 | **60^c^** | 38 | 30 | **83** | **36** | 3 |
| A17L_61-70_ | RTLLGLILFV | Tg mice | -8 | -2 | 8 | 15 | 52 | 7 | 7 | -1 | 0 |
| A17L_81-90_ | ILMIFISSFL | Tg mice | 16 | 0 | 0 | **48** | -75 | **43** | -5 | 34 | 2 |
| A17L_138-146_ | QIFNIISYI | Tg mice | 4 | -3 | 2 | -2 | -63 | -2 | -2 | -1 | 0 |
| A46R_142-150_ | GLFDFVNFV | Tg mice | -8 | -3 | 10 | 12 | 38 | 2 | 3 | -3 | 0 |
| B6R_108-116_ | LMYDIINSV | Tg mice | -6 | -3 | 3 | -2 | 23 | -2 | 10 | -4 | 0 |
| B14R_327-335_ | HVDGKILFV | Tg mice | -8 | -3 | -2 | -2 | 85 | -2 | 48 | 16 | 0 |
| D12L_251-259_ | RVYEALYYV | Tg mice | -6 | -3 | 3 | **97** | 27 | 15 | 8 | **29** | 2 |
| F11L_196-204_ | FLIVSLCPT | Tg mice | -1 | -3 | 5 | 0 | -15 | 0 | -13 | 6 | 0 |
| G7L_250-258_ | YLPEVISTI | Tg mice | -8 | -2 | -2 | 5 | 38 | 0 | 23 | 18 | 0 |
| H3L_184-192_ | SLSAYIIRV | Tg mice | -11 | -3 | 2 | -2 | 0 | -2 | 25 | 3 | 0 |
| I1L_211-219_ | RLYDYFTRV | Tg mice | 21 | -3 | 3 | 2 | 18 | 2 | **115** | 23 | 1 |
| M1L_374-383_ | IIIPFIAYFV | Tg mice | **74** | 0 | 21 | **32** | 71 | **20** | **220** | **52** | 5 |
| C7L_250-258_ | KVDDTFYYV | Human + Tg mice | -1 | 2 | 2 | **232** | -7 | **123** | -7 | 6 | 2 |
| G5R_18-26_ | ILDDNLYKV | Human + Tg mice | **108** | **33** | 2 | **680** | -37 | **383** | -10 | **151** | 5 |

^a^ Donors tested in the original screen by Oseroff *et al.* [9].

^b^ Net SFC/10^6^ PBMCs.

^c^ Bolded SFC values indicate a positive response (as defined by SFC/10^6^ cells ≥ 20, stimulation index ≥ 2.0, and p-value ≤ 0.05.
